# Management of Ischemia and Necrosis Following Nonsurgical Rhinoplasty With Hyaluronic Acid: A Case Report

**DOI:** 10.7759/cureus.103290

**Published:** 2026-02-09

**Authors:** Andrea Tedesco, Anna Clara C Commanducci Silva, Roberta G Neves, Ithala L Oliveira Parpinelli, Francine F Posser Ribeiro, Sabrina C Pacheco, Alan Ferreira, Antony P Barbosa

**Affiliations:** 1 Dental Clinic, Faculty of Dentistry, Federal University of Rio de Janeiro, Rio de Janeiro, BRA; 2 Private Practice, Andrea Tedesco Institute, Rio de Janeiro, BRA; 3 Research and Development, Antony Barbosa Institute, Belo Horizonte, BRA; 4 Pharmacy, Pontifical Catholic University of Minas Gerais, Belo Horizonte, BRA

**Keywords:** hyaluronic acid, hyaluronidase, ischemia, necrosis, nonsurgical rhinoplasty

## Abstract

Nonsurgical rhinoplasty with hyaluronic acid (HA) is minimally invasive but may result in severe vascular complications such as ischemia and necrosis. This case report describes a 30-year-old female patient who developed ischemia and early necrosis of the nasal tip after HA injection. Treatment was initiated approximately 72 hours after the injection, and management included multipoint hyaluronidase, massage and warm compresses, low-level red laser therapy, systemic antibiotic and corticosteroid administration, in addition to hyperbaric oxygen therapy and photodynamic therapy. Regression of ischemia was observed on the first day after treatment initiation, and complete re-epithelialization occurred within 26 days. Early diagnosis and multimodal management centered on hyaluronidase favored ischemia reversal and wound healing, minimizing sequelae.

## Introduction

The use of hyaluronic acid (HA) fillers for nonsurgical rhinoplasty has increased markedly over the past decade (approximately 2015-2025), driven by their immediate aesthetic impact, minimal downtime, and high levels of patient satisfaction [1]. Despite these advantages, the nasal region remains one of the most anatomically challenging and high-risk areas for HA injection. Its dense and variable vascular network, particularly the branches of the dorsal nasal and angular arteries, creates a setting in which even small deviations in technique may result in significant vascular compromise [2].

Vascular events, although infrequent, represent among the most feared complications in aesthetic practice due to their potential for skin necrosis, permanent scarring, and, in rare cases, visual loss [2,3]. Current evidence indicates that these adverse outcomes typically arise from inadvertent intravascular injection or extrinsic compression of arterial branches, leading to reduced perfusion and subsequent tissue ischemia [3]. Early recognition of warning signs, such as disproportionate pain, livedo reticularis, or progressive pallor, remains critical for mitigating tissue damage.

Current consensus guidelines and expert recommendations emphasize immediate and repeated administration of high-dose pulsed hyaluronidase as the primary treatment for HA-related vascular occlusion, ideally initiated as soon as ischemia is suspected and repeated until reperfusion is achieved [4]. Adjunctive therapies, including warm compresses, low-level laser therapy, hyperbaric oxygen, and photodynamic therapy (PDT), have been reported as supportive measures that may assist reperfusion and promote tissue healing [5-9]. However, the timely integration of these modalities in real-world scenarios, particularly when patients present late or receive inadequate initial management, remains a challenge.

In this context, the present case highlights the reversal of advanced ischemia following nonsurgical rhinoplasty through a multimodal treatment approach, demonstrating that meaningful tissue recovery may still occur even when intervention is not performed within the early ideal treatment window.

## Case presentation

A 30-year-old female nurse with no significant past medical history, known allergies, or previous aesthetic complications presented for nonsurgical nasal reshaping using HA filler (Rennova® Ultra Deep, Panaxia Ltd., Lod, Israel). She reported good overall health, denied tobacco use, and had previously undergone minimally invasive facial procedures without adverse events. The injection session, performed on June 7, 2024, was described as uneventful, with the immediate postoperative appearance being satisfactory to both the practitioner and the patient. Micropore tape was applied for structural support.

On the following day, after self-removal of the micropore tape, the patient experienced sudden-onset nasal pain, bleeding, and a small area of superficial tissue detachment at the nasal tip. She initially managed these symptoms at home, but progressive worsening prompted her to seek care at an emergency department approximately 24 hours post-procedure. At that visit, she received systemic corticosteroids and sedatives; however, no vascular-targeted intervention such as hyaluronidase was administered. Over the next 48 hours, the patient noted increasing pain, dark discoloration, and spreading areas of pallor and livedo across the nasal tip and dorsum.

**Figure 1:**
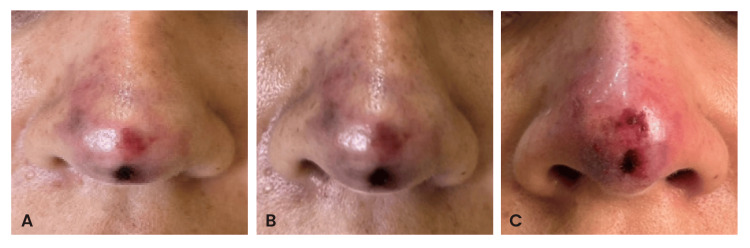
Initial presentation on day 3 after nonsurgical rhinoplasty showing livedo, pallor, and early necrosis of the nasal tip and dorsum (A-C), with ischemic progression extending toward the glabella. Images were provided and published with the patient’s authorization in accordance with the informed consent form.

Seventy-two hours after the procedure, she presented to a specialized aesthetic complications clinic. Physical examination revealed marked ischemia affecting the nasal tip and dorsum, extending toward the glabella, with a central necrotic plaque surrounded by livedoid and ulcerated borders (Figure 1). Capillary refill was markedly delayed, and soft tissue induration was evident on palpation. No systemic symptoms such as fever, malaise, or lymphadenopathy were reported. Although no laboratory or imaging studies were deemed necessary at this stage, the clinical presentation was consistent with advanced HA-related vascular occlusion.

Given the extent of ischemia and the delayed presentation, a multimodal rescue protocol was immediately initiated. The treatment plan prioritized rapid enzymatic degradation of residual HA, restoration of perfusion, and mitigation of further tissue injury. High-dose hyaluronidase (1500 IU; Mesoestetic®, Barcelona, Spain) was injected across multiple strategic points (~20 IU per point) along the presumed courses of the facial and angular arteries. Each infiltration was followed by vigorous manual massage, warm compresses to promote vasodilation, and red-light low-level laser therapy (6 J) to stimulate microcirculation. This cycle was repeated three times at one-hour intervals.

She also received intramuscular ceftriaxone 1 g for infection prophylaxis and a single dose of dexamethasone (systemic corticosteroid) to reduce inflammation and secondary edema. By the end of the first treatment day, perfusion had improved, capillary refill was restored, and progressive regression of ischemia became evident.

The patient was discharged on oral amoxicillin/clavulanate for seven days and referred for hyperbaric oxygen therapy (HBOT), with five sessions planned to enhance tissue oxygenation and support angiogenesis. On June 12, persistent induration at the nasal tip prompted an additional hyaluronidase session complemented by warm compresses and low-level laser therapy. Her plastic surgeon prescribed tadalafil for three days to support vasodilation and a topical cicatrizing ointment (Regencel®; Cristália, Brazil) to aid re-epithelialization.

Between June 14 and June 25, the patient underwent four sessions of PDT using 0.1% methylene blue combined with red-light activation. This modality was selected for its antimicrobial and photobiomodulatory effects, which may support wound healing in ischemic tissue. Progressive contraction of the ulcerated areas, resolution of necrotic tissue, and formation of healthy granulation tissue were observed throughout the course of therapy.

**Figure 2:**
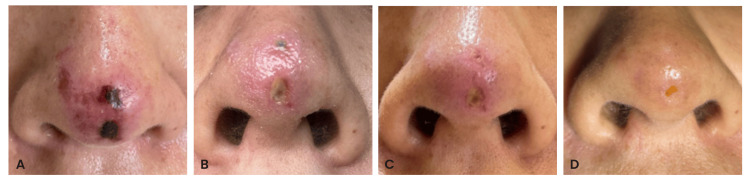
Clinical progression following multimodal treatment, demonstrating gradual reperfusion, reduction of ischemic areas, and re-epithelialization from day 3 to day 26 (A-D). Images were provided and published with the patient’s authorization in accordance with the informed consent form.

By July 3, 2024, complete wound closure had been achieved, with no evidence of secondary infection, scarring, deformity, or functional impairment (Figure 2). The achieved outcome was consistent with expectations for a late-presenting vascular occlusion managed with an intensive multimodal approach.

## Discussion

Vascular complications associated with nonsurgical rhinoplasty, although uncommon, remain among the most serious adverse events in aesthetic practice due to the intricate vascular architecture of the nasal region and its anastomotic connections with the ophthalmic circulation [2,3]. While reported incidence varies widely, vascular obstruction may be underrecognized in routine practice, particularly when early signs are subtle or transient [10]. Prompt identification of warning features - disproportionate pain, blanching, livedo reticularis, cool skin, and delayed capillary refill - is essential, as treatment delay increases the risk of necrosis and long-term sequelae [3].

Practical clinical algorithm (early recognition and immediate steps) includes (1) stop injection immediately at the first suspicion of vascular compromise; (2) assess perfusion (capillary refill, skin temperature, extent of livedo/pallor) and document findings; (3) initiate immediate high-dose pulsed hyaluronidase with multi-point infiltration across the affected angiosome and along suspected arterial pathways, repeating at short intervals until reperfusion signs improve; (4) support reperfusion with vigorous massage and warm compresses; and (5) escalate care when ischemia persists or necrosis is evolving, including referral to a complications, such as focused service and consideration of adjuvant modalities. Adjunctive measures should support, but not delay, prompt enzymatic degradation of HA.

The cornerstone of management remains early, repeated high-dose hyaluronidase, which degrades HA and may restore microvascular flow [4]. Most published reports describe the best outcomes when treatment is initiated within hours after symptom onset. The present case is notable because targeted vascular management began 72 hours after injection. Yet, meaningful ischemia reversal and complete re-epithelialization were still achieved, supporting the concept that aggressive intervention may remain beneficial beyond the ideal therapeutic window when clinical signs suggest persistent compromise.

Adjunctive therapies may support tissue recovery once reperfusion efforts are underway. Warm compresses and massage promote vasodilation and microcirculatory flow, while low-level laser therapy is used to modulate inflammation and support tissue repair [6]. HBOT may assist ischemic tissue through improved oxygen delivery and support of angiogenesis [7,9]. PDT using methylene blue has been reported as a supportive strategy with antimicrobial and photobiomodulatory properties that may aid wound healing in compromised tissue [8]. A second novel aspect of this report is the combined, sequential use of HBOT and PDT as adjuncts after delayed hyaluronidase-based rescue, which is less commonly detailed in the existing rhinoplasty occlusion literature. While this single case cannot establish efficacy, it highlights a plausible adjunctive pathway for late-presenting ischemia that warrants further study.

This case also reflects real-world challenges: inconsistent early recognition, delayed access to hyaluronidase, and initial management that did not address the underlying vascular pathophysiology. These factors reinforce the need for practitioner training, patient education on warning signs, and readily available emergency protocols. When implemented promptly and systematically, evidence-informed rescue strategies may improve prognosis, even in advanced presentations.

Overall, this report reinforces that vascular occlusion after HA rhinoplasty, while potentially devastating, may still be managed with a structured approach centered on high-dose pulsed hyaluronidase and supported by carefully selected adjunctive therapies. The patient’s recovery without functional or aesthetic sequelae underscores the importance of early recognition and immediate action, while also highlighting the potential value of multimodal escalation in delayed cases.

## Conclusions

This case highlights how prompt recognition of vascular compromise and the coordinated use of a multimodal rescue strategy can significantly alter the course of an otherwise severe complication following nonsurgical rhinoplasty. Even with a delayed presentation, the combination of repeated high-dose hyaluronidase, thermal vasodilation, low-level laser therapy, hyperbaric oxygen, and PDT contributed to full tissue recovery and prevented lasting deformity. The favorable outcome reinforces the importance of practitioner preparedness, patient education, and clear emergency protocols to ensure timely intervention. Ultimately, this report underscores that, while vascular events remain a serious risk, structured and comprehensive management can restore tissue viability and preserve both function and aesthetic integrity. Prospective studies and complication registries will be essential to better define optimal treatment algorithms and evaluate the reproducibility of these outcomes.
